# Double-blind, placebo-controlled, randomized phase II study of TJ-14 (hangeshashinto) for gastric cancer chemotherapy-induced oral mucositis

**DOI:** 10.1007/s00280-014-2440-x

**Published:** 2014-03-21

**Authors:** Toru Aoyama, Kazuhiro Nishikawa, Nobuhiro Takiguchi, Kazuaki Tanabe, Motohiro Imano, Ryoji Fukushima, Junichi Sakamoto, Mari S. Oba, Satoshi Morita, Toru Kono, Akira Tsuburaya

**Affiliations:** 1 Department of Surgery, Miura City Hospital, Miura, Japan; 2 Department of Surgery, Osaka General Medical Center, Osaka, Japan; 3 Department of Gastroenterological Surgery, Chiba Cancer Center, Chiba, Japan; 4 Department of Gastroenterological and Transplant Surgery, Hiroshima University, Hiroshima, Japan; 5 Department of Surgery, Kinki University Faculty of Medicine, Osaka-Sayama, Japan; 6 Department of Surgery, Teikyo University School of Medicine, Tokyo, Japan; 7 Tokai Central Hospital, Kagamigahara, Japan; 8 Department of Biostatistics and Epidemiology, Yokohama City University, Yokohama, Japan; 9 Advanced Surgery Center, Sapporo Higashi Tokushukai Hospital, Sapporo, Japan; 10 Shonan Kamakura General Hospital, Kamakura, Japan

**Keywords:** Oral mucositis, Hangeshashinto (TJ-14), Chemotherapy, Gastric cancer

## Abstract

**Background:**

Hangeshashinto (TJ-14, a Kampo medicine), which reduces the level of prostaglandin E2 and affects the cyclooxygenase activity, alleviates chemotherapy-induced oral mucositis (COM). We conducted a randomized comparative trial to investigate whether TJ-14 prevents and controls COM in patients with gastric cancer.

**Methods:**

We randomly assigned patients with gastric cancer who developed moderate-to-severe oral mucositis (CTCAE v4.0 grade ≧1) during any cycle of chemotherapy to receive either TJ-14 or a placebo as a double-blind trial. The patients received a placebo or TJ-14 for 2–6 weeks according to the chemotherapy regimen from the beginning of the next course of chemotherapy. The primary end point was the incidence of grade ≧2 oral mucositis in the protocol treatment course, and the secondary end points were the time to disappearance of oral mucositis and the incidence of adverse events.

**Results:**

Following the key opening of the blinding protocol, we analyzed 91 eligible patients (TJ-14: 45, placebo: 46) using a “per protocol set” analysis. The incidence of ≧grade 2 COM was 40.0 % in the TJ-14 group and 41.3 % in the placebo group (*p* = 0.588). The median duration of ≧grade 2 COM was 14 days in the TJ-14 group and 16 days in the placebo group (*p* = 0.894). Meanwhile, the median duration of any grade of COM was 9 days in the TJ-14 group and 17 days in the placebo group among the patients who developed grade 1 symptoms during the screening cycle [hazard ratio 0.60; 95 % CI (0.23–1.59), *p* = 0.290].

**Conclusions:**

Although TJ-14 treatment did not reduce the incidence of ≥2 COM in the patients who developed mucositis during chemotherapy for gastric cancer, a trend was observed in which TJ-14 reduced the risk of COM in the patients who developed grade 1 COM during the screening cycle. Further, phase III studies with a larger sample size are needed to clarify the protective effects of TJ-14 for COM.

## Introduction

Gastric cancer is the second most frequent cancer-related cause of death after lung cancer [1]. Chemotherapy is one of the most important modalities for treating advanced gastric cancer as well as curatively resected cancers in the adjuvant setting. Numerous chemotherapy regimens have been used in cases of operable or inoperable gastric cancer [2–5]. Although several studies have shown that chemotherapy improves and prolongs survival, it often causes severe toxicity, seriously compromising the patient’s quality of life and precluding the continuation of the treatment.

Oral mucositis is a common toxicity associated with cytotoxic chemotherapy used in the gastric cancer treatment. In pivotal phase III trials of chemotherapy for gastric cancer, the incidence of all grades of chemotherapy-induced oral mucositis (COM) was observed to be 6.3–32 % [4–8]. COM results in severe discomfort, impairing the patient’s ability to eat, swallow, and talk, and has an indirect effect on tumor outcomes, as its presence often necessitates the unfavorable modification of anticancer therapy, such as breaks in the administration of chemotherapy or dose reduction in the chemotherapy regimen [9–11]. One factor associated with COM exacerbation is the activation of the cyclooxygenase pathway, which mediates ulcer formation and pain via the upregulation of pro-inflammatory prostaglandins. Indeed, Richard et al. demonstrated, after having enlisted 20 patients treated with chemotherapy drugs and performing a biopsy of the oral mucosa in each case, a statistically significant increase in the number of endothelial cells in the oral mucosa with nuclear factor-kappa B (NF-κB) and cyclooxygenase 2 (COX-2) expressions in the postchemotherapy treatment period compared to that observed in the pretreatment period. The expression of COX-2 in these cells represents the initial sign of the inflammatory cascade that determines the production of prostaglandins and further tissue damage. COX-2 is also upregulated by NF-κB, which plays an important role in the inflammatory process [12]. COM invariably requires treatment with systemic analgesics, adjunctive medications, physical therapy, and psychological therapy in addition to oral care [13]. Treatment guidelines developed by the Multinational Association of Supportive Care in Cancer and the International Society for Oral Oncology have been published; however, they also highlighted the need for a higher level of evidence [14]. Although a range of interventions have been developed to prevent and treat COM, a more rational approach is warranted [11].

Hangeshashinto (TJ-14) is a traditional Japanese medicine containing 7 herbal crude drugs. Seven herbal crude drugs are as follows; Pinelliae tuber, Scutellariae Radix, Glycyrrhizae Radix, Zizyphi Fructus, Ginseng Radix, Zingiberis Processum rhizoma, and Coptidis rhizome [15–17]. TJ-14 is prescribed in Japan to treat inflammatory diarrhea, gastritis, and stomatitis. Recently, Kono et al. [18] found that TJ-14 was effective as a gargle therapy for the treatment of COM in a pilot clinical study and a randomized, placebo-controlled clinical trial. TJ-14 has been demonstrated to directly inhibit PGE2 production in human gingival fibroblasts and reduce the PGE2 content in the colon in several animal models of diarrhea using anticancer drugs, cholera toxin, or castor oil, resulting in the amelioration of inflammatory damage [19–22]. It has also been reported that some ingredients of TJ-14 inhibit PGE2 production and/or the COX-2 expression [23–32]. Phenylpropanoids, such as [6]-shogaol and [6]-gingerol, flavonoids, such as wogonin, baicalein, and baicalin, and isoquinoline alkaloids, such as berberine, are well established to possess an anti-PGE2 activity via various particular mechanisms.

Considering these clinical and biochemical study findings, in the present study, the efficacy of TJ-14 in the prevention and/or treatment of COM was investigated in a randomized, double-blind, placebo-controlled clinical trial of patients receiving chemotherapy for gastric cancer.

## Materials and methods

### Study design

A prospective, multi-institutional, randomized, double-blind, placebo-controlled phase II trial was performed in patients receiving chemotherapy for gastric cancer in Japan. Patients who developed CTCAE v4.0 ≧grade 1 oral mucositis during the screening cycle of chemotherapy were considered eligible for inclusion in this study. The eligible patients were centrally randomized to receive either TJ-14 or a placebo during their next cycle of chemotherapy. The patients were stratified according to age, chemotherapy regimen, institution, and previous treatment for oral mucositis before randomization in a 1:1 ratio. A specially made and prepared matched placebo was utilized to confirm blinding.

The primary objective of this study was to determine the efficacy and safety of TJ-14 compared with the placebo. The primary end point was the incidence of ≥grade 2 oral mucositis, and the worst oral mucositis grade observed throughout the protocol therapy was assessed. As for severity, the worst grade observed on the day of the medication was evaluated instead of the mean circadian change. The secondary end points were the time to disappearance of oral mucositis and the incidence of adverse events.

### Ethics

The study data and informed consent were obtained in accordance with the Declaration of Helsinki, and the study protocol was approved by the Ethics Review Board of each institution. The institutions where ethics was obtained were as follows: Kanagawa Cancer Center, Osaka General Medical Center, Chiba Cancer Center, Hiroshima University, Kinki University, Teikyo University, Toyonaka Municipal Hospital, Hiroshima City Asa Hospital, Prefectural Aichi Hospital, Kochi University, Minoh City Hospital, National Hospital Organization Nagoya Medical Center, Shizuoka General Hospital, Nagoya City University, and Osaka-Kita-Teishin Hospital. All patients were given a written explanation of the study protocol and provided their written informed consent before participating.

### Inclusion and exclusion criteria

Patients 20 years of age or older who were undergoing chemotherapy for gastric cancer were considered eligible for this study. Patients who developed moderate-to-severe oral mucositis (CTCAE v4.0 grade ≧1) during any cycle of chemotherapy (S-1, paclitaxel, irinotecan, cisplatin, etc.) were asked to be enrolled in the study. All participants were required to have a “good” performance status (i.e., scores of 0 or 1 on the Eastern Cooperative Oncology Group performance status scale). Patients with any of the following characteristics were not eligible for the study: use of Kampo medicine within 2 weeks before registration and a history of severe hypersensitivity (allergy) to any medicine containing antiphlogistics, analgesics, opioids, or steroids. Patients with serious constipation and pregnant or lactating females were excluded from the study. Any other medical conditions that made a patient unsuitable for inclusion in the study according to the opinion of the investigator were also considered to be exclusion criteria for this study.

### Chemotherapy

Gastric cancer chemotherapy was administered according to the protocols of each treatment, and the administration of each agent was described in case report form. Patients enrolled in this study received the following chemotherapy.

- Group A: S-1 monotherapy. S-1: The treatment regimen consisted of 6-week cycles in which 80 mg/m^2^ per day was given for 4 weeks followed by 2-week rest for adjuvant setting and 5-week cycles in which 80 mg/m^2^ per day for 3 weeks followed by 2-week rest for advanced gastric cancer patients
- Group B: S-1 plus cisplatin. S-1: 80 mg/m^2^ oral administration (p.o.) daily for 21 days, every 5 weeks. Cisplatin: 60 mg/m^2^ intravenous drip (d.i.v.) day 8, every 5 weeks.
- Group C: S-1 plus paclitaxel. S-1: 80 mg/m^2^ p.o. daily on days 1–14 of 3 weeks cycle. Paclitaxel: 50 mg/m^2^ d.i.v. days 1, 8 every 3 weeks.
- Group D: paclitaxel. Paclitaxel: 80 mg/m^2^ d.i.v. days 1, 8, 15, every 4 weeks.
- Group E: S-1 plus docetaxel. S-1: 80 mg/m^2^ p.o. daily on days 1–14 of 3 weeks cycle. Docetaxel: 40 mg/m^2^ d.i.v. days 1 every 3 weeks.
- Group F: docetaxel. Docetaxel: 60 mg/m^2^ d.i.v. days 1 every 3 weeks.
- Group G: CPT-11 plus cisplatin. CPT-11: 60 mg/m^2^ d.i.v. days 1 every 2 weeks. Cisplatin: 30 mg/m^2^ d.i.v. days 1 every 2 weeks.
- Group H: CPT-11. CPT-11: 150 mg/m^2^ d.i.v. days 1 every 2–3 weeks

### Study drug

Both TJ-14 and the placebo were administered at a dose of 2.5 g/three times per day (for a total daily dose of 7.5 g). The placebo formulation matched the texture, flavor, and other characteristics of the active drug. The patients were advised to dissolve 2.5 g of TJ-14 or the placebo in 50 ml of drinking water and rinse their oral cavity with the solution three times daily for 10 s. The test drug was administered from the first day to final day of the protocol treatment course. After the protocol treatment course, TJ-14 was administrated for one course, as much as possible. The patients followed the oral care instructions throughout the treatment period before the next course of chemotherapy began. No other prophylactic mouthwashes or treatments for mucositis were allowed in this clinical trial.

### Study assessment

The signs and symptoms of oral mucositis were assessed by the investigator during the screening cycle. The CTCAE v4.0 grading (Table 1) was used to assess the severity of oral mucositis. The time to healing of oral mucositis was defined as the period from the start date of the protocol treatment or the date of onset of oral mucositis to the date when all oral mucositis symptoms disappeared. If all oral mucositis symptoms fail to disappear within the study treatment period, the observation shall be continued until symptom disappearance. Additionally, the patients reported their own ability to eat solid foods. Safety was assessed throughout the study using physical examinations, including inspection of the oral tissue, hematology and serum chemistry laboratory tests, and adverse event reporting. Any adverse event, whether related or unrelated to the study drug, was reported with the date and time of onset, severity, pattern, action taken, and outcome. If the adverse event had not resolved at the time the case report forms were collected, a follow-up report was provided a later date. If no follow-up report was provided, the investigator had to provide justification. All adverse events were followed until they either resolved or the investigator determined that the event was no longer clinically significant.

**Table 1:** Severity of oral mucositis

Grade 1	Asymptomatic or mild symptoms; Intervention not indicated
Grade 2	Moderate pain; not interfering with oral intake; Modified diet indicated
Grade 3	Severe pain; Interfering with oral intake
Grade 4	Life-threatening consequence; Urgent intervention indicated
Grade 5	Death

Common Terminology Criteria for Adverse Events (CTCAE) Version 4.0

### Statistical analysis

The eligible patients were randomly assigned on a 1:1 ratio to receive either TJ-14 or the placebo. After checking patient eligibility, randomization was carried out centrally at the data center using dynamic randomization with main prognostic factors, including the chemotherapy regimen (postoperative adjuvant chemotherapy, unresectable metastatic/recurrent lesions), presence/absence of previous treatment of oral mucositis, age (≥60 years, <60 years), and institution.

Assuming an incidence of grade 2 or worse COM of 10 % in the TJ-14 group and 35 % in the placebo group, a sample size of 42 for each group was estimated to have at least 80 % power under a significance level of two-sided 10 %. Therefore, in order to account for possible dropouts, a target sample size of 90 patients was required.

The difference in the incidence of grade 2 or worse COM between the groups and its 90 % confidence interval was calculated. Comparisons were made using the chi-squared test. The baseline characteristics were compared using the chi-squared test for categorical variables and the Wilcoxon test for continuous variables. The Kaplan–Meier method, log-rank test, and Cox proportional hazard regression model were used to assess the time to healing among the patients with COM. A hazard ratio (HR) smaller than 1 indicated that TJ-14 accelerated the healing of COM. The frequencies of adverse events were compared using Fisher’s exact test. All *p* values were two-sided. The statistical analyses were performed using the SAS software package for Windows, release 9.3 (SAS Institute, Cary, NC).

## Results

### Patients

Of the patients receiving chemotherapy for gastric cancer, 91 who developed CTCAE v4.0 ≧grade 1 oral mucositis during the screening cycle and provided informed consent were randomized to either the TJ-14 (*n* = 45) or placebo (*n* = 46) group. The baseline demographics and disease characteristics of the per protocol set (PPS) population are shown in Table 2. A total of 61.5 % of the subjects were male, and 38.5 % of the subjects were female; the median age was 68 years (range 36–89 years). All patients had histologically confirmed gastric adenocarcinoma. There were no disparities between the two PPS randomized groups. The majority of patients received S-1 adjuvant (48.4 %) or S-1-based doublet (22.0 %) regimens, and the treatment groups were balanced for the chemotherapy regimen (Table 2). No patients received radiation therapy or molecular targeting agents before enrollment. No patients were enrolled in the study if there was any clinical evidence of another active oral mucosal disease at baseline.

**Table 2:** Patient characteristics of the TJ-14 and placebo groups

Treatment	Placebo (*N* = 46)	TJ-14 (*N* = 45)	*p* value
*Sex*			
Male	28 (60.9 %)	28 (62.2 %)	0.895
Female	18 (39.1 %)	17 (37.8 %)	
*Age*			
Median	67.5	68.0	0.648
Range	42.0–89.0	36.0–84.0	
*PS*			
0	38 (82.6 %)	39 (86.7 %)	0.855
1	5 (10.9 %)	4 (8.9 %)	
2	3 (6.5 %)	2 (4.4 %)	
*Status*			
Adjuvant	21 (45.7 %)	23 (51.1 %)	0.602
Advanced	25 (54.3 %)	22 (48.9 %)	
*Oral care (patients)*			
+	3 (6.5 %)	2 (4.4 %)	0.664
−	43 (93.5 %)	43 (95.6 %)	
*Oral care (institution)*			
+	11 (23.9 %)	7 (15.6 %)	0.317
−	35 (76.1 %)	38 (84.4 %)	
*Chemotherapy at the time of registration*			
S-1	19 (42.2 %)	25 (55.6 %)	0.490
S-1 + CDDP	3 (6.7 %)	5 (11.1 %)	
S-1 + DTX	6 (13.3 %)	1 (2.2 %)	
S-1 + PTX	2 (4.4 %)	3 (6.7 %)	
DTX	1 (2.2 %)	1 (2.2 %)	
PTX	3 (6.7 %)	2 (4.4 %)	
CPT-11 + CDDP	1 (2.2 %)	2 (4.4 %)	
CPT-11	3 (6.7 %)	1 (2.2 %)	
5-FU + CDDP	0 (0 %)	1 (2.2 %)	
other	7 (15.6 %)	4 (8.9 %)	

*CDDP* Cisplatin, *PTX* Paclitaxel, *DTX* Docetaxel, *5*-*FU* 5-fluorouracile

### Incidence and duration of COM

**Fig. 1:**
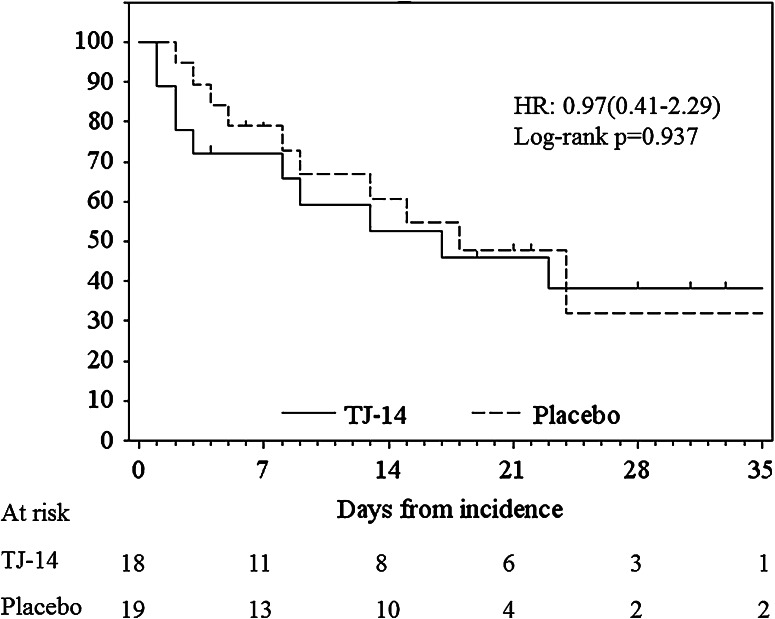
Duration of ≧grade 2 chemotherapy-induced oral mucositis in the treatment group

The incidence of ≧grade 2 COM was 40 % (18 patients) in the TJ-14 group and 41.3 % (19 patients) in the placebo group, and there was no significant difference between the two groups (*p* = 0.588); the primary end point was not met in this study. More, when comparing the duration of ≧grade 2 COM between the two treatment groups, there was not significantly difference (HR 0.97 (0.41–2.29) log-rank *p* = 0.937) (Fig. 1).

**Fig. 2:**
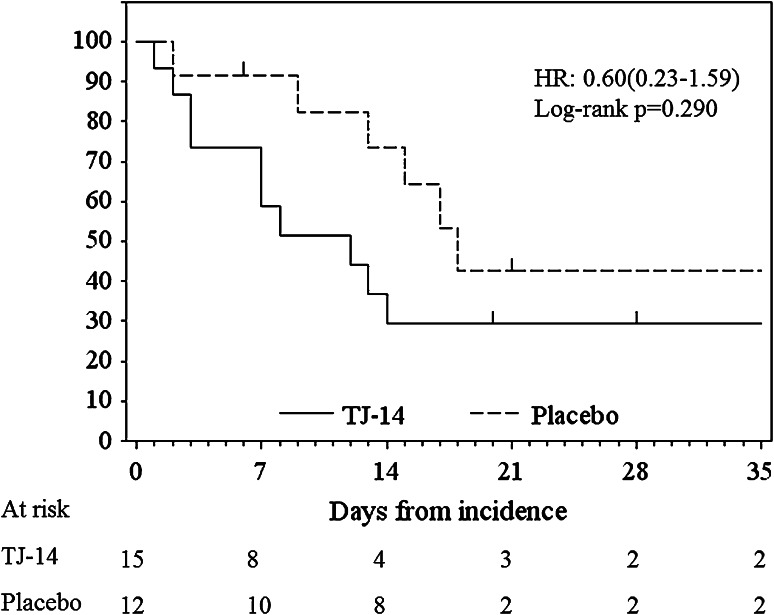
Duration of any grade of chemotherapy-induced oral mucositis in the patients who developed grade 1 oral mucositis during the screening cycle

However, among the patients who developed Grade 1 COM during the screening cycle, the median duration of any grade of COM was 9.0 days in the TJ-14 group and 17.0 days in the placebo group [HR 0.598; 95 % CI (0.226–1.585), *p* = 0.290] (Fig. 2). Treatment with TJ-14 reduced the duration of any grade of COM compared with the placebo.

### Chemotherapy treatment failure during the protocol treatment

Chemotherapy treatment failure was observed in 26.7 % (12 patients) of the subjects in the TJ-14 group and 21.7 % (10 patients) of the subjects in the placebo group. For most chemotherapy regimens, there were no significant differences with regard to the incidence of the treatment failure.

### Safety

Hematological, blood biochemistry, and non-hematological toxicities were analyzed. The most commonly reported treatment-related adverse events were anorexia, a change in PS, nausea, neutropenia, and diarrhea, all of which typically occur in cancer patients receiving cytotoxic chemotherapy (Table 3). The majorities of these events were mild to moderate in severity and considered to be unrelated to the study drug.

**Table 3:** Hematological and biochemical toxicities observed during the treatment

	≥Grade 1			≥Grade 2		
	TJ-14 (*N* = 45)	Placebo (*N* = 46)	*p* value	TJ-14 (*N* = 45)	Placebo (*N* = 46)	*p* value
*Hematological toxicity*						
Leucopenia	5 (11 %)	8 (17 %)	0.39	0 (0 %)	1 (2 %)	0.32
Neutropenia	7 (16 %)	7 (15 %)	0.96	3 (7 %)	4 (9 %)	0.72
Hemoglobin	37 (82 %)	40 (87 %)	0.53	13 (29 %)	8 (17 %)	0.19
Platelet	4 (9 %)	6 (13 %)	0.53	0 (0 %)	1 (2 %)	0.32
T-Bilirubin	3 (7 %)	4 (9 %)	0.72	0 (0 %)	0 (0 %)	1.00
AST	2 (4 %)	2 (4 %)	0.98	0 (0 %)	0 (0 %)	1.00
*Non*-*hematological toxicity*						
Anorexia	18 (40 %)	19 (41 %)	0.90	8 (18 %)	4 (9 %)	0.20
Nausea	7 (16 %)	9 (20 %)	0.62	2 (4 %)	2 (4 %)	0.98
Vomiting	3 (7 %)	2 (4 %)	0.63	0 (0 %)	1 (2 %)	0.32
Diarrhea	5 (11 %)	4 (9 %)	0.70	0 (0 %)	1 (2 %)	0.32
Constipation	3 (7 %)	5 (11 %)	0.48	0 (0 %)	1 (2 %)	0.32
Peripheral neuropathy	1 (2 %)	1 (2 %)	0.99	0 (0 %)	1 (2 %)	0.32
Lassitude	3 (7 %)	3 (7 %)	0.99	0 (0 %)	1 (2 %)	0.32
Hand-foot syndrome	0 (0 %)	1 (2 %)	0.32	0 (0 %)	1 (2 %)	0.32
Skin reaction	2 (4 %)	0 (0 %)	0.15	0 (0 %)	1 (2 %)	1.00
Dysgeusia	2 (4 %)	1 (2 %)	0.54	1 (2 %)	1 (2 %)	0.99
Edema	1 (2 %)	1 (2 %)	0.99	0 (0 %)	1 (2 %)	0.32
Change in PS	8 (18 %)	9 (20 %)	0.83	2 (4 %)	3 (7 %)	0.66

*AST* aspartate aminotransferase

## Discussion

To date, this randomized trial is the first evaluation of the use of TJ-14 to treat COM in patients with gastric cancer in a prospective placebo-controlled randomized phase II study. The primary purpose of this study was to prove the effects of TJ-14 in reducing the incidence of ≧grade 2 oral mucositis. The incidence of oral mucositis of ≧grade 2 was 40.0 % in the TJ-14 group and 41.3 % in the placebo group in the overall study population. Therefore, treatment with TJ-14 did not exhibit any effect with regard to reducing the frequency of grade 2 events or the duration of grade 2 chemotherapy-induced oral mucositis in gastric cancer patients receiving fluorinated pyrimidine-based chemotherapy. Why did this trial not meet its primary objective? The most likely reason is that the dose reduction of chemotherapy performed before the administration of TJ-14 treatment may have affected the incidence and duration of COM. Generally, among patients who developed ≧grade 2 COM before being entered into this study, the physicians may have been inclined to stop or postpone the original chemotherapy and reduce the dose at the time of the next chemotherapy cycle, which was exactly the time of study treatment and observation [33]. The incidence of oral mucositis of ≧grade 2 was 36.4 % in the patients who received chemotherapy dose reduction and 42.0 % in the patients who did not receive dose reduction. With regard to the incidence of toxicity in this study, 36 patients developed CTCAE v4.0 ≧grade 2 oral mucositis during the screening cycle. Among these patients, seven received dose reduction before the protocol cycle. The median duration of ≧grade 2 oral mucositis was 10.0 days in the patients treated with prophylactic dose reduction and 16.0 days in the patients treated without prophylactic dose reduction. There was a significant difference between the two groups (*p* = 0.034). It has been reported that the COM was depending on the dose and type of chemotherapy [34, 35]. Coleman et al. [36] evaluated 116 women with measurable metastatic breast cancer participated in a randomized phase II study of single-agent liposomal pegylated doxorubicin given either as a 60 mg/m^2^ every 6 weeks (ARM A) or 50 mg/m^2^ every 4 weeks (ARM B) schedule. They found that the adverse event profiles of the two schedules were distinctly different, and mucositis was more common with ARM A (35 % CTC grade 3/4 in ARM A, 14 % in ARM B). More, Elting et al. [37] retrospectively analyzed 599 patients who developed chemotherapy-induced oral mucositis. They found that a reduction in the dose of the next cycle of chemotherapy was twice as common after cycles with mucositis as it was after cycles without mucositis (23 vs. 11 %; *p* ≤ 0.0001). Taking these findings into consideration, dose reduction in the chemotherapy regimen may have been a key issue improving the incidence and/or the duration of COM. We assume that the effects of TJ-14 in oral mucositis may be less prominent due to the use of chemotherapy dose reduction just before the experimental cycles. Taking these findings into consideration, dose reduction in the chemotherapy regimen may have been a key issue improving the incidence and duration of COM. We assume that the effects of TJ-14 in oral mucositis may be less prominent due to the use of chemotherapy dose reduction just before the experimental cycles.

A borderline significant difference, however, was observed in the patients who developed ≧grade 1 COM at the time of screening. The median duration of any grade of oral mucositis was 9.0 days in the TJ-14 group and 17.0 days in the protocol treatment cycle group. Treatment with TJ-14 reduced the duration of any grade of oral mucositis compared with the placebo. In patients with grade 1 COM before the experimental cycle, it is presumed that the physicians may not have reduced the chemotherapy dose. Therefore, most of the patients who developed COM of grade 1 were not influenced by dose reduction of chemotherapy. These results suggest that the effects of TJ-14 would have been more prominent if chemotherapy dose reduction had not been performed before the experimental cycles. As mentioned above, it has been previously reported that TJ-14 exerts an anti-inflammatory effect by suppressing the levels of lipopolysaccharide-induced IL-6 and IL-8, and cyclooxygenase (COX)-1 and COX-2 [38, 39], in a dose-dependent manner [40]. Further studies are needed to clarify the exact mechanisms underlying these observations.

In conclusion, this trial did not show a beneficial effect of TJ-14 in reducing the incidence of chemotherapy-induced oral mucositis as the primary end point, likely due to the use of dose reduction before the experimental cycles, which was not prohibited by the study protocol. In the patients with >grade 1 COM at the screening cycle, an obvious reduction in the risk of COM (HR 0.60) was demonstrated. In this regard, the addition of TJ-14 to chemotherapy regimens may have shortened the duration of oral mucositis when no dose reduction was performed before the administration of the experimental agents. A further analysis may lead to a better interpretation of the study results by examining subgroups that will benefit from TJ-14 treatment. A more definitive design in a future trial of TJ-14 for chemotherapy-induced oral mucositis is needed to eliminate the influence of arbitrary dose reduction based on the discretion of the individual physician.
